# Connaissances attitudes et pratiques du personnel des salons de coiffures sur l'infection à VIH à Lomé Togo

**DOI:** 10.11604/pamj.2019.32.217.16421

**Published:** 2019-04-30

**Authors:** Julienne Noude Teclessou, Bayaki Saka, Abravi Emefa Sabli, Abla Séfako Akakpo, Abas Mouhari-Touré, Koussake Kombate, Palokinam Pitche

**Affiliations:** 1 Service de Dermatologie-Vénérologie CHU Lomé; Faculté des Sciences de la Santé, Université de Lomé, Lomé, Togo; 2 Service de Dermatologie-Vénérologie, CHU Campus Lomé, Lomé, Togo; 3 Service de Dermatologie-Vénérologie CHU Kara, Faculté des Sciences de la Santé, Université de Kara, Kara, Togo

**Keywords:** Salons de coiffure, VIH/sida, Lomé, Hair salons, HIV/AIDS, Lomé

## Abstract

**Introduction:**

le but de cette étude était de décrire les connaissances, attitudes et pratiques du personnel des salons de coiffure sur l'infection à VIH à Lomé.

**Méthodes:**

il s'est agi d'une étude descriptive, dont la population d'étude était constituée par les patrons et les apprentis des salons de coiffure de la préfecture d'Agoè-nyivé à Lomé entre le 1^er^ octobre 2016 et le 31 mars 2017. Les différents paramètres étudiés étaient les données sur la connaissance générale du VIH, les attitudes et pratiques sur le VIH/sida dans les salons de coiffure.

**Résultats:**

au total, 203 patrons et apprentis présents dans les 68 salons de coiffure des préfectures d'Agoè-nyivé ont été enquêtés. L'infection à VIH/ sida était connue par tous (100%) les participants et 79,3% d'entre eux la définissaient comme étant une infection sexuellement transmissible. Le port de tablier ou de gants avant certains gestes de coiffure était fait dans respectivement 33(51,5%) et 35(48,5%) salons de coiffures. Aussi, dans 60(88,2%) salons de coiffure, une décontamination des objets tranchants était faite avant leur usage. Cependant, l'alcool était le désinfectant le plus utilisé par la majorité (89,3%) du personnel des salons de coiffure. Aussi, l'ébullition pendant en moyenne 7 minutes était réalisée par 79,8% des coiffeurs. En cas d'accident d'exposition au sang, 69,6% du personnel des salons de coiffure faisaient un nettoyage du site exposé avec de l'alcool.

**Conclusion:**

cette étude a montré que les coiffeurs/coiffeuses et leurs apprentis ont de bonnes connaissances sur l'infection à VIH/sida, sur ses modes de transmission et ses moyens de prévention dans les salons de coiffure. Cependant, certaines pratiques dont les méthodes de désinfections du matériel souillé et la conduite en cas d'accidents d'exposition au sang étaient mauvaises.

## Abstract (en)

**Introduction:**

this study aims to describe the knowledge, attitude and practices of hairdressers about HIV infection in Lomé.

**Methods:**

we conducted a descriptive study whose population included hair salon owners and apprentices in the Agoè-Nyivé prefecture, Lomé, between October 1 2016 and March 31 2017. The different parameters studied were data on the general knowledge about HIV, attitudes and practices about HIV/AIDS in the hair salons.

**Results:**

a total of 203 owners and apprentices were interviewed in the 68 hair salons in the Agoè-Nyivé prefecture. The study population (100%) knew about HIV/AIDS. Among the participants, 79.3% of them defined it as a sexually transmitted infection. Gloves and aprons were worn before some hairstyling gestures in 33 (51.5%) and 35 (48.5%) hair salons respectively. Moreover, in 60 (88.2%) hair salons, sharp instruments were disinfected prior to their use. However, alcohol was the disinfectant the most commonly used by the majority of the staff of hairdressing salons (89.3%). Moreover, boiling for an average of 7 minutes was performed by 79.8% of hairdressers. In the event of blood exposure accident, 69.6% of staff of hair salons cleaned the exposed site with alcohol.

**Conclusion:**

this study shows that hairdressers and their apprentices have good knowledge about HIV infection/AIDS, its modes of transmission and prevention means in hair salons. However, it highlights that the disinfection of soiled equipment and the behaviour of hairdressers and their apprentices in the case of blood exposure accident are sometimes improper.

## Introduction

Près de 40 ans après sa découverte, l'infection par le VIH constitue toujours un défi pour notre humanité. Ainsi, la communauté internationale et les pays se sont engagés à mettre fin à l'épidémie du SIDA en 2030 [1]. Selon le rapport ONUSIDA de juillet 2017, on comptait 36,7 millions de personnes vivant avec le VIH (PVVIH) dans le monde dont 1,8 million de personnes nouvellement infectées [2]. Au Togo, selon l'enquête démographique et de santé, la prévalence du VIH dans la population générale était de 2,5% en 2014 [3]. La lutte contre l'épidémie implique une bonne prévention de celle-ci. Les salons de coiffure sont les lieux où l'on utilise plusieurs instruments tranchants (lames, aiguilles, ciseaux, tondeuses...) comme outils de travail. Ces outils de travail, lorsqu'ils sont mal entretenus peuvent être source de contamination de plusieurs virus dont le VIH et les virus des hépatites. La connaissance des moyens de prévention du VIH dans les salons de coiffure, a fait objet de peu d'études en Afrique [4], et aucune n'a concerné le Togo. Le but de cette étude était de décrire les connaissances, attitudes et pratiques du personnel des salons de coiffure à Lomé sur l'infection à VIH.

## Méthodes

Il s'est agi d'une étude descriptive, qui a été menée dans les salons de coiffure de la préfecture d'Agoè-nyivé à Lomé pendant une période de 6 mois, du 1^er^ octobre 2016 au 31mars 2017. Le Togo est un pays d'Afrique de l'ouest qui comptait environ 7 500 000 d'habitants en 2014 [5]. On y distingue six régions sanitaires du nord vers le sud: la région des savanes, la région de la Kara, la région centrale, la région des plateaux, la région maritime et la région de Lomé commune. La préfecture d'Agoè-nyivé constitue l'une des 8 préfectures de la région de Lomé commune. Elle a une superficie de 139 km^2^ pour une population de 399,411 habitants, et est composée de 5 cantons. Les salons de coiffure présents dans cette préfecture sont organisés en syndicat et inscrits à la chambre du commerce et des métiers (CCM). Il s'agit d'une organisation syndicale incluant les coiffeurs, couturières, et autres métiers d'art. Tous les salons de coiffure homme, dame et mixte inscrits à la CCM ont été sélectionnés. La population d'étude était constituée par les responsables et les apprentis de ces salons de coiffure présents lors de l'enquête. Au Togo, la majorité des métiers d'art dont la coiffure se fait par apprentissage et sont sanctionnés par un examen organisé par les structures gouvernementales. La collecte des données a été faite à l'aide d'un questionnaire préétabli et d'un guide d'observation après l'obtention de l'accord des responsables de la CCM et le consentement éclairé des participants.

**Les données sociodémographiques:** âge, sexe, niveau d'instruction.

**La connaissance sur le VIH/sida:** définition, les sources d'information sur le VIH/sida, les modes de transmission et les moyens de prévention du VIH/sida en général et dans les salons de coiffure en particulier.

**Les attitudes et pratiques de prévention du VIH/sida dans les salons de coiffure:** le dépistage du VIH dans les trois mois précédant l'enquête, l'espace de mobilité dans les salons de coiffure. Cette mobilité est dite restreinte lorsqu'elle est inférieure à 6 mètres carrés, moyennement large lorsqu'elle est comprise entre 6 et 12 mètres carrés et large lorsqu'elle est supérieure à 12 mètres carrés. Les mesures d'hygiène d'une part (existence d'une source d'eau courante, du savon et/ou de désinfectant, de boite pour isoler les matériaux tranchants (lames, aiguilles)), la décontamination des matériels et la conduite en cas d'accident d'exposition au sang (AES) ont été également étudiées. L'analyse des données a été faite avec le logiciel Epi info7. La comparaison des variables quantitatives a été faite par la méthode Chi-2 et le test de Fisher. Le seuil de significativité a été fixé à 5%.

**Approbation éthique:** le protocole d'étude a été soumis et a été approuvé par le comité de bioéthique du ministre de la santé. Un consentement éclairé écrit et verbal a été obtenu auprès de la chambre des métiers et des arts de la préfecture et auprès des répondants avant le début de l'enquête. Les personnes interrogées ont été dûment informées de la manière dont l'étude sera menée l'étude.

## Résultats

Nous avons inclus au total 68 salons de coiffure dont 47 salons de coiffure pour femmes (69,1%), 18 salons de coiffure pour hommes (26,5%) et 03 salons mixtes (4,4%). Dans ces salons de coiffure, 203 personnels présents au moment de l'enquête avaient donné leur consentement pour participer à l'enquête. Il s'agit de 138 (68,0%) apprentis et 65 (32,0%) coiffeurs/coiffeuses. L'âge moyen des participants était de 25 ± 7,38 ans (extrêmes: 19 et 45 ans) et le sex-ratio (M/F) de 3,3. La majorité (94,6%) des participants avait au moins le niveau d'instruction primaire. L'infection à VIH/sida était connue par tous (100%) les participants et tous affirmaient avoir suivi des séances de sensibilisation sur le VIH/sida par l'intermédiaire des médias (télévisions, radios) ou lors des séances de sensibilisation de masse. Aussi, 79,3% des participants définissaient l'infection à VIH comme étant une infection sexuellement transmissible (IST) (Tableau 1). La définition de l'infection à VIH comme étant IST était significativement associée au niveau d'instruction des enquêtés (p=0,01).

**Tableau 1:** connaissance, transmission et prévention du VIH en générale et dans les salons de coiffure en particulier

Questions	Proposition juste	%	proposition fausse	%
Le VIH/sida existe-il?	203	100	0	0
**Définition du VIH**				
Le VIH est une maladie sexuellement transmissible	161	79,3	42	20,7
Le VIH est une maladie liée au travail du sexe	41	20,2	162	79,8
Le VIH est une maladie lié à un mauvais sort jeté	14	6,9	189	93,1
Je ne sais pas	4	2,0	199	98,0
**Transmission du VIH en générale**				
Le VIH se transmet par les rapports sexuels non protégé	202	99,5	1	0,5
Le VIH se transmet par blessure par objet tranchant souillé	200	98,5	3	1,5
Le VIH se transmet de la mère à l'enfant pendant la grossesse, l'accouchement ou l'allaitement	166	81,8	37	18,2
**Le VIH peut se transmet dans les salons de coiffure par**				
Aiguille, lame, ciseaux, rasoirs	178	87,7	25	12,3
Tondeuses	60	29,6	143	70,4
Peigne	27	13,3	176	86,7
Bigoudi	6	3,0	197	97,0
Mèche	4	2,0	199	98,0
**Prévention du VIH en générale**				
Rapport sexuel protégés	200	98,5	3	1,5
Eviction contact avec des objets souillés de sang	188	92,6	15	7,4
Abstinence	144	70,9	59	29,1
Fidélité	142	70,0	61	30,0
**La prévention du VIH dans les salons de coiffure peut se faire par**				
Désinfection/stérilisation du matériel après usage	199	98,5	4	2,0
Changement du matériel (uniquement les lames) après usage	170	83,8	33	16,2
Port de gants	140	69,0	63	31,0
Lavage du matériel à eau savonneuse	73	36,0	130	64,0

Les rapports sexuels non protégés et les plaies par objets tranchants souillés constituaient les voies de transmission les plus connues (respectivement 99,5% et 98,5% des enquêtés). Les objets évoqués comme étant à risque de transmettre le VIH dans les salons de coiffure étaient les lames, ciseaux, aiguilles, et rasoirs (87,7%), suivi des tondeuses (29,6%) (Tableau 1). Les rapports sexuels protégés constituaient le moyen de prévention du VIH/sida cité par 98,5% des enquêtés; suivi de l'éviction du contact avec objets souillés et l'abstinence (respectivement 92,6% et 70,9%). La majorité des participants (98,5%) évoquaient la désinfection/stérilisation du matériel tranchant (aiguilles, ciseaux, tondeuses) et le changement du matériel (lames) après usage (83,8%) comme moyen de prévention du VIH dans les salons de coiffure (Tableau 1). La plupart des enquêtés (79,8%) avaient déjà réalisés au moins une fois le test de dépistage du VIH et 49,3% l'avaient réalisé durant les 03 derniers mois précédant notre enquête. Parmi les participants qui n'avaient jamais réalisé le test de dépistage, aucun motif n'était évoqué par 48,8% et 24,4% avaient peur de connaitre leur statut.

L'espace de mobilité était moyennement large dans 55,9% des salons de coiffure (Tableau 2). Soixante (88,2%) des 68 salons de coiffure disposaient d'une poubelle et d'une boite à objet tranchant. Respectivement 50 (73,5%) et 30 (44,1%) salons de coiffure avaient une source d'eau courante et un lavabo (Tableau 2). Le port de gants avant certains gestes de coiffure était fait dans 40 (60,3%) salons de coiffure. Aussi, dans 60 (88,2%) salons de coiffure, une désinfection des objets tranchants était faite avant leur usage. L'alcool et les dérivés chlorés notamment l'eau de javel étaient les désinfectants les plus utilisés par les personnels des salons de coiffure (respectivement 89,3% et 63,7%). Ces désinfectants étaient utilisés seul ou en association. La désinfection consistait en un nettoyage instantané du matériel de coiffure avec du coton imbibé de ces désinfectants. Elle était appliquée principalement aux ciseaux, rasoirs, tondeuses, aiguilles et peigne. Les lames étaient changées après premier usage dans 91,2% des salons de coiffure. Le comportement adopté en cas d'un accident d'exposition au sang (AES) était le nettoyage du site exposé par de l'alcool uniquement (69,6%) ou en association avec d'autres conduites (Tableau 2). En revanche, seulement 6,4% avaient recours à une consultation médicale dans les 48 heures suivant un AES.

**Tableau 2:** attitudes et pratiques du personnel des salons de coiffure face au VIH/sida

Questions	Oui	%	Non	%
**Espace de mobilité dans les salons (n=68)**				
Restreint	12	17,7	56	82,3
Moyennement large	38	**55,9**	30	44,1
Très large	18	26,5	50	73,5
**Présence de matériel de désinfection dans les salons de coiffure (n=68)**				
Savon	67	98,50	1	1,50
Autres désinfectant	62	91,20	6	8,80
Poubelle et boite à objet tranchant	60	88,20	8	11,80
Source d'eau	50	73,53	27	39,70
Lavabo	30	44,10	38	55,90
**Moyens de prévention utilisés dans les salons (n=68)**				
Désinfection d'objets tranchant avant leur usage	60	88,20	8	11,80
Changement du matériel (uniquement les lames) après usage	62	91,2	6	8,8
Port de gants avant de coiffer	41	60,30	27	39,70
Utilisation de gants de ménage ou de tablier avant nettoyage des locaux	34	50,0	34	50,0
**Conduite en cas d'accident d'exposition au sang (n=203)**				
Nettoyage alcool	117	69,6	86	42,4
Consultation médicale (dans les 48 heures suivant l'AES)	13	6,4	190	93,6
Rien	12	5,9	191	94,1
Lavage à l'eau et au savon, suivi de rinçage	8	3,9	195	96,1
Désinfection par des dérivés iodés	5	2,5	198	97,5
Désinfection par l’eau de javel	4	1,0	201	99,0

## Discussion

La principale difficulté rencontrée au cours de notre enquête était liée à la méfiance des responsables des salons de coiffures étant donné que l'enquête concernait leur vie professionnelle. Cette étude a montré que les coiffeurs/coiffeuses et leurs apprentis ont de bonnes connaissances sur l'infection à VIH notamment sa définition (79,3% la définissaient comme étant une maladie sexuellement transmissible), ses modes de transmission (87,7% évoquaient la transmission par les aiguilles, lames, ciseaux, rasoirs dans les salons de coiffure) et ses moyens de prévention dans les salons (83,8% évoquaient le changement du matériel après usage). On notait également certaines bonnes attitudes et pratiques dans les salons notamment la présence de savon et autres désinfectants dans respectivement 98,5% et 91,2% des salons de coiffure et la désinfection de certains objets après usage (88,2%). Cependant certaines pratiques dont principalement la conduite en cas d'AES n'était pas bonne puisque 69,6% du personnel de ses salons de coiffures nettoyaient les sites avec de l'alcool et seul 6,4% avaient recours à une consultation médicale.

L'infection à VIH/sida était connue par tous les participants de notre étude. La connaissance du VIH/sida était également retrouvée chez 100% des participants dans une étude en 2009 au Nigéria [6]. Les sources d'informations étaient constituées des médias (radio, télévision). Les médias constituent donc une source d'informations importante dans la lutte contre le VIH/sida, quelques soit le niveau d'instruction des populations. Respectivement 99,5 % et 98,5% des enquêtés avaient évoqué les rapports sexuels non protégés et les objets à risque contaminés comme mode de transmission du VIH/sida. Bien que nous n'ayons pas évalué la connaissance approfondie des participants, ces résultats montrent une bonne connaissance des modes de transmission et de prévention du VIH en général. La majorité (87,7%) des participants avaient cités les aiguilles, lames, ciseaux et rasoirs comme étant des objets à risque de transmission du VIH/sida dans les salons de coiffure. Cependant, seul 29,7% avaient cité les tondeuses comme objet à risque de transmission. Ceci pourrait s'expliquer par le fait que 69,1% des salons de coiffure étaient des salons pour femmes ou les tondeuses sont rarement utilisées. L'espace de mobilité était restreint et moyennement large dans respectivement 17,7% et 55,9% des salons de coiffure, constituant un facteur de risque élevé de transmission du VIH. En effet, lors de la manipulation de certains objets tranchants (lame, aiguille) dans les salons de coiffures peu larges, ces objets peuvent accidentellement piquer une tierce personne d'où la transmission du VIH par voie sanguine. La désinfection du matériel (ciseaux, rasoirs, tondeuses, aiguilles et peigne) après usage était réalisée dans 88,2% des salons de coiffure et le changement des lames après usage dans 91,2% des salons de coiffure.

En 2014 au Pakistan, seul 60,3% de coiffeurs utilisaient une nouvelle lame entre les clients [7]. Aussi, en 2012 en Ethiopie, respectivement 94,1% et 78,7% des coiffeurs évoquaient l'importance de la stérilisation et de la désinfection comme moyen de prévention des infections [8]. Nos résultats sont également proches de ceux de Oyedunni *et al.* au Nigéria où 91,2% des coiffeurs avaient cité la décontamination des instruments comme moyen de prévention du VIH [6]. Bien que la désinfection du matériel soit faite dans la majorité des salons de coiffure, les désinfectants les plus utilisés dans notre étude étaient l'alcool (89,3%) et l'eau de javel (63,7%). En 2012 en Ethiopie, l'usage de l'alcool et de l'eau de javel était retrouvé chez respectivement 46,7% et 45,7% des participants [8]. Le taux élevé d'utilisation de l'alcool comme désinfectant dans notre étude constitue cependant une mauvaise pratique dans ces salons étant donné le pouvoir fixateur de l'alcool. Les dérivés chlorés dont l'eau de javel sont connus comme ayant une efficacité sur le VIH. Mais le matériel à désinfecter doit être plongé dans la solution chlorée pendant une durée d'au moins 5 minutes [9]. Ceci n'est pas le cas dans les salons de coiffure de notre étude ou la désinfection se fait par nettoyage instantané du matériel avec la solution désinfectante. Aussi, l'eau de javel utilisée dans les salons de coiffure n'est pas à une concentration précise. Il serait donc important de remettre à niveau les responsables des salons de coiffures quant à la pratique de la désinfection des objets à risque. En cas d'AES, l'alcool (69,6%) était utilisé pour nettoyer la plaie et seul 6,4% avaient recours à une consultation médicale dans les 48 heures suivant l'accident. En 2010 en Italie, une étude avait montré que 82,9% des coiffeurs avaient évoqué l'usage d'antiseptique en cas de blessure des clients [10]. Nos résultats confirment une mauvaise pratique en cas d'AES dans les salons de coiffure.

## Conclusion

Cette étude nous permet de conclure à une bonne connaissance du VIH/sida, de ses moyens de prévention et des modes de transmission chez le personnel des salons de coiffure de la préfecture d'Agoè-Nyivé à Lomé. Cependant, les attitudes et pratiques doivent être améliorées. Malgré le bon relais des informations sur le VIH/sida par les médias, il est important d'organiser des séances de formation sur les attitudes et pratiques enfin de limiter les risques de contamination de l'infection dans les salons de coiffures.

### Etat des connaissances actuelles sur le sujet

- Le VIH reste à nos jours un problème majeur de santé publique en Afrique subsaharienne;
- Peu d'études se sont intéressées au VIH dans les salons de coiffure en Afrique subsaharienne et aucune étude n'a porté sur le sujet au Togo.

### Contribution de notre étude à la connaissance

- Connaissance des modes de prévention et de transmission du VIH dans les salons de coiffures;
- Attitudes et pratiques du personnel des salons de coiffure face au VIH.

## Conflits d’intérêts

Les auteurs ne déclarent aucun conflit d'intérêts.

## Remerciements

Les auteurs remercient les responsables de la chambre des métiers et arts de la préfecture d'Agoé-Nyévé pour avoir facilité leur accès aux salons de coiffure.
